# Long reads: their purpose and place

**DOI:** 10.1093/hmg/ddy177

**Published:** 2018-05-14

**Authors:** Martin O Pollard, Deepti Gurdasani, Alexander J Mentzer, Tarryn Porter, Manjinder S Sandhu

**Affiliations:** 1 Human Genetics - Wellcome Sanger Institute, Hinxton, Cambridge, UK; 2 University of Cambridge - Department of Medicine, Addenbrookes Hospital, Box 157, Hills Road, Cambridge, UK; 3 Wellcome Centre for Human Genetics, Roosevelt Drive, Oxford, UK

## Abstract

In recent years long-read technologies have moved from being a niche and specialist field to a point of relative maturity likely to feature frequently in the genomic landscape. Analogous to next generation sequencing, the cost of sequencing using long-read technologies has materially dropped whilst the instrument throughput continues to increase. Together these changes present the prospect of sequencing large numbers of individuals with the aim of fully characterizing genomes at high resolution. In this article, we will endeavour to present an introduction to long-read technologies showing: what long reads are; how they are distinct from short reads; why long reads are useful and how they are being used. We will highlight the recent developments in this field, and the applications and potential of these technologies in medical research, and clinical diagnostics and therapeutics.

## When Short Reads Are Not Enough

DNA is an extraordinarily compact storage medium, so small that developing ways to decode the sequence encoded in these molecules has been a topic of research for many years. The first method developed for sequencing DNA, often known as Sanger sequencing (1), was a low throughput process that detected bases by incorporation into a template strand, sequencing fragments of DNA up to 1000 bp long. The breakthrough allowing sequencing at scale finally came with the advent of next generation sequencing (NGS) technology, which employed massively parallel reactions for high throughput. While these technologies have been able to capture sequence from the majority of the genome and have found utility in the study of disease, their short reads and lack of contextual information has limited their utility in genome assembly and in resolving complex and repetitive regions of the genome.

**Figure 1.**
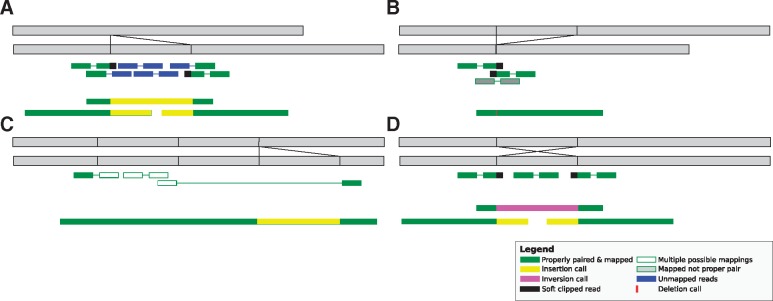
Behaviour of reads around genomic events. (**A**) Large insertion: short reads at the edge of the variant are be soft-clipped. Reads within the insertion will be either unmapped or mapped incorrectly. Large reads will either span the insertion or have enough context to be marked as inserted sequence. (**B**) Large deletion: short reads spanning the deletion may be mismapped or only have one of the reads marked as mapped because the reference measured length indicates the insert size deviates from the expected distribution. Long reads will span the gap but most will have enough context to call the deletion. (**C**) Copy number variation: where the read-length exceeds the length of the CNV region reads will map correctly. Shorter reads may be collapsed and show up as increased depth in a pileup or be marked as mapping poorly. (**D**) Inversion: reads will either be represented as a primary alignment with an inverted supplementary or manifest as soft clipping around the edge of the inversion with a reduction in depth where reads span the edge of the inversion.

The incremental improvements in read-length that this generation of technology can yield is one of diminishing returns. Thus, to achieve substantial gains in mapping, assembly and phasing one must consider technology that provides an order of magnitude increase in read-length (2). Practically also, there are many important problems in genetics where a short read of DNA (<1000 base pairs) is insufficient (Table 1 and Fig. 1).

**Table 1.** Advantages and applications of long-read sequencing

Limitations of short read data	Applications and advantages of long-read sequencing
Access to high GC content regions Resolution of complex regions of the genome (e.g. MHC^a^) Repetitive regions where short reads will not map uniquely Systematic context-specific error modes Structural variation, and large segmental duplications Paralogous regions of the genome Resolution of phase (read-based phasing)	De novo assembly from long reads to span the low complexity and repetitive regions, to create accurate assemblies (3). Targeted sequencing of complex genomic and paralogous regions and resolution of phase for clinical applications e.g. HLA^b^ typing, ADPKD^c^ (4). Transcriptomics, allowing full length sequencing of isoforms and examination of splicing (5). Detection of structural variants (e.g. segmental duplications, gene loss and fusion events) Single molecule sequencing allows examination of clonal heterogeneity of pathogens, and immunogenic cells Long-range characterization of methylation patterns

a MHC: Major histocompatibility complex.

b HLA: Histocompatibility leucocyte antigen.

c ADPKD: Autosomal-dominant polycystic kidney disease.

Key to achieving high quality results with all long-read technologies is the use of high molecular weight DNA as a starting material. The utility of these methods depends on a long DNA fragment size, with DNA damage and fragmentation limiting the quality of data obtained. Specific protocols for DNA extraction such as the agarose gel protocol for BioNano are ideal to maximize yield from these methods.

## Long-Read Technologies

### Single molecule real time sequencing

**Figure 2.**
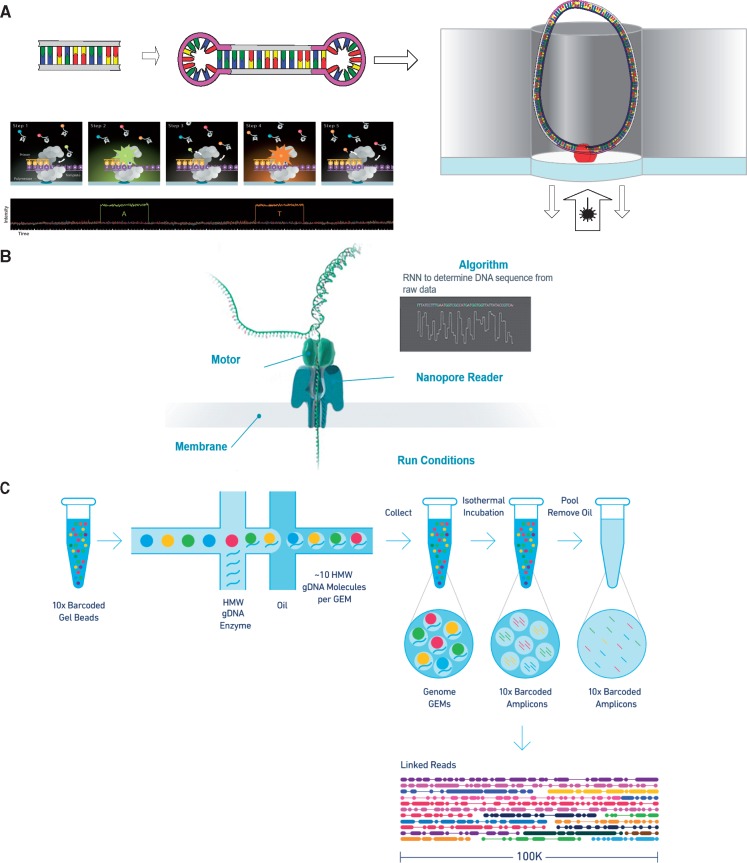
Long-read sequencing technologies. (**A**) PacBio SMRT sequencing. Double stranded DNA is first sheared and size selected to the desired length and then sequencing adaptors are annealed. The adaptors are bound to a sequencing primer and strand displacing polymerase which adheres to the bottom of a well containing a zero mode wave guide. Following a pre-extension period where the polymerase reaction is run in the dark, the fragment is illuminated with a laser and as each base in the sequencing solution is incorporated, the fluorophore is detected and the polymerase reaction displaces it, giving a time and intensity signal which is converted into a base call. (**B**) Oxford Nanopore Technology passes the DNA molecule through a nanopore attached the flow cell surface membrane. As each base of the DNA molecule passes through the pore changes to the current passing through the pore are detected and converted into a signal. The signal detected is passed to a recurrent neural network (RNN) which converts it into base calls. (**C**) 10X Genomics Chromium technology works by means of an emulsion droplet technology, where gel beads are mixed with high molecular weight genomic DNA and an enzyme. Within each gel bead DNA is sheared and barcoded, creating fragments which can then be sequenced with Illumina sequencing. The presence of the chromium barcode then provides a mapper or assembler with linked-reads, allowing the relative spatial position of the fragments to be estimated *Components of figure reproduced with permission from Pacific Biosciences, Oxford Nanopore Technologies and 10X Genomics.*

The first long-read sequencing technology to achieve a widespread deployment is the single molecule real time (SMRT) sequencing technology from Pacific Biosciences (PacBio). The SMRT system implemented in their Sequel and RS- II platforms uses a massively parallel system of polymerases each bound to a single molecule of target DNA that has been circularized with a pair of hairpin sequencing adaptors (the SMRTbell) (Fig. 2A). Incorporation of labelled bases by a polymerase on the template strand causes fluorescence. The resulting signal is detected by a CCD camera via a zero-mode waveguide (6,7), yielding a combination of signal and time series information. Reads produced by this technology typically peak at 100 Kbp in length and a typical N50 on recent polymerases is ∼20 Kbp.

One complication of SMRT sequencing is the high error rate of this process relative to short read sequencing, at 11–14% depending on polymerase and chemistry. However, this error mode is stochastic (by contrast with other technologies), and can be mitigated by repeated measurements of the sequence. With PacBio sequencing, this is carried out by repeated forward and reverse sequencing passes over the circularized SMRTbell molecule (Fig. 2A). Adaptor sequences can be removed from the generated sequence to provide enough subreads to generate a highly accurate consensus of each molecule. This process is known as circular consensus sequencing and has been shown to reduce basecalling error substantially (8) whilst also enabling the strand specific calling of base modifications in unamplified DNA (9). When long DNA fragments are sequenced, these may not be parsed more than once in the SMRTbell; in this case, increasing coverage and then calling a consensus across reads can also achieve a reduced error rate; a method frequently used in polishing assemblies (10).

### Oxford Nanopore Technologies

The next successful single molecule technology to hit the market was that produced by Oxford Nanopore Technologies (ONT) (11). This technology is based on passing a single strand of DNA through a nanopore with an enzyme attached, and measuring changes in the electrical signal across the pore (Fig. 2B). The signal is then amplified and measured to determine the bases that passed through. As the pore holds several bases at a time (typically 5-mers), overlapping k-mers that cause changes in raw current must be inferred and used to make base calls, a process which can be error prone. By measuring the shape of the molecule passing through the pore ONT not only reads the sequence of the DNA but like SMRT is also able to detect base modifications (12). However, unmodelled base modifications and systematic DNA context-specific errors (13) currently limit the utility of the technology.

Oxford Nanopore MinION technology heralds the promise of a pocket size sequencer, with reads from ONT that can stretch into the hundreds of kilobases with appropriate DNA preparation, and megabase long reads that have been observed when a large number of flow cells have been used. There appears to be no intrinsic read-length limit for ONT, other than the size of DNA fragments. Recent improvements in technology, library preparation and throughput have allowed the first human line sequenced on the MinION (GM12878) earlier this year (14). This study generated ultra-long reads (>800 Kbp), and suggested that addition of modest coverage with ultra-long-read sequencing to existing assemblies may substantially improve resolution of contigs and haplotypes. While the error rate is comparable to SMRT sequencing, a component of the error is systematic and context-specific, limiting the ability to correct this by increasing coverage (13) and requiring polishing with other technologies instead.

ONT has developed a distinct strategy to mitigate stochastic error on their platform, focusing on the way that the template strand passes through the pore. ONT cannot simply circularize the DNA. Instead, both the template and complement strands of the DNA molecule are joined by a hairpin loop during library prep (2D) or tethered in such a way (1D^2^) to allow sequential forward and reverse strand sequencing. Combining these data greatly enhances accuracy and reduces random error.

The use of nanopores as a nucleic acid sequencing technology is not entirely exclusive to ONT; at least one similar but distinct competing technology is also under development by Roche.

### 10X Genomics Chromium system

An alternative to the aforementioned single molecule sequencing methods is the 10X Genomics Chromium system. Whilst this is not technically a long-read sequencing technology, it is an important member of this ecosystem and can solve similar problems such as mapping, phasing and assembly (Fig. 2C). Chromium has lower cost compared to ONT and SMRT because of the use of the nearly ubiquitous Illumina short reads in its sequencing process.

The basis of this technology (15) is the barcoding of large fragments of DNA (preferably >100 Kbp) in an initial digital droplet polymerase chain reaction (PCR) step. In each droplet, a single fragment is both sheared and then tagged with a semi-unique molecular barcode (Fig. 2C). The resulting fragments are then sequenced like any other Illumina library. The barcode allows for determination of the relative spatial orientation of the tags, and allows phasing and assembly of contigs by combining information across multiple tags (15,16). Additionally, because the data provide spatial orientation across the genome, it is possible to use it to scaffold data from other methods (17).

### Allied technologies

Allied technologies associated with long-read sequencing such as: optical mapping, HiC and similar have been used to enhance the final results from sequencing. Optical mapping technologies such as BioNano Irys and Saphyr label DNA and then image the labelled DNA to generate genome maps. These genome maps are used to scaffold contigs produced by assembly (18) and also to discover large (>500 bp) structural variants and inversions. HiC can be used to assay chromosomal conformation and is particularly useful in assigning assembled sequences to chromosomes (19).

## The Utility of Long-Read Technology: Recent Developments

### High resolution genome assemblies

**Figure 3.**
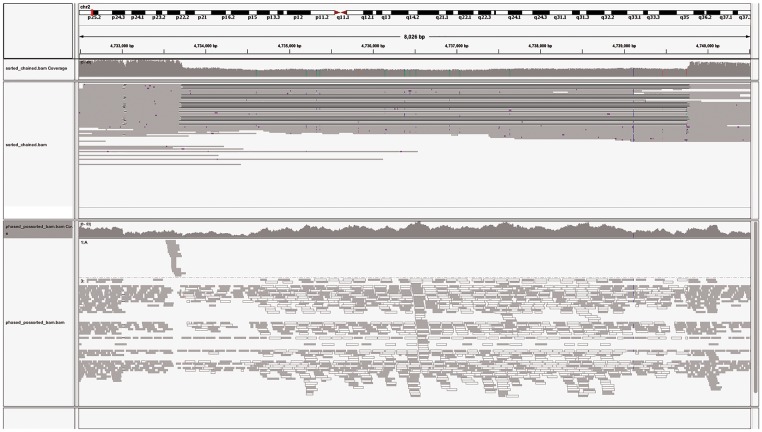
Long reads span and call variations that short reads cannot. IGV (http://software.broadinstitute.org/software/igv/home) image of (top) PacBio reads from a sample sequenced as part of the GDAP project. The reads span a 6 kb heterozygous LINE-1 element deletion and show clear depth variation. Illumina (bottom) reads from the same sample unable to be clearly mapped around the deletion with reads in white indicating where reads were unable to be uniquely mapped.

Accurate assemblies of the genomes of organisms are crucial to understanding organismal diversity, speciation, evolution of species and the impact of genomic diversity on health and disease. The current human genome reference GRCh38 has been assembled from the DNA of multiple donors, and represents a mosaic of haplotypes. However, several studies have suggested that existing human reference genomes may not fully reflect the diversity of global human populations, and may be biased towards diversity in European populations (20–22). This has important implications for human basic and medical research. Assembling the human genome has involved extensive curation with clone-based assembly methods and Sanger sequencing. Long-read technologies provide a high throughput platform for characterization of genomes through highly contiguous assemblies (Fig. 3).

The early long-read platforms produced reads that were only a few kilobases long with a high per-base cost; however, they quickly carved a niche in the creation and finishing of assemblies. These long reads could close gaps in genomes by spanning the low complexity regions that would otherwise require many costly YAC, BAC and fosmid clones to be created and sequenced. Thus, many of the early tools such as PBJelly were focused on gap closure (23,24). The high per-base error rate also required new assembly algorithms, and new tools were created to polish the final assembly with Illumina reads to eliminate basecalling error (12). Clone based assembly methods were not eliminated entirely either as they provided useful spatial context, but long reads provided a new way to sequence clones in a high throughput manner (25).

Long-read sequencing methods have contributed to platinum quality reference sequences such as NA12878 (14,26) and the haploid sequences CHM1 (27) and CHM13 (28), as well filling many gaps in the human reference (18,25,29). Of particular note are the first Chinese (18) and Korean (25) human reference genomes which have been created to answer questions about population-specific sequence. These sequences have resulted in highly contiguous assemblies, closing a high proportion of gaps in the human genome. These have led to discovery of population-specific sequences, demonstrating the need for further assemblies from non-European population groups. Recently, higher coverage sequencing (∼60×) of two haploid genomes has also been used to identify substantial structural variation, the vast majority of which have not been recovered from sequencing using NGS technologies (28). Characterization of high resolution population-specific reference genomes from initiatives such as the Genome in a Bottle (GIAB) (30) and the Genome Diversity in Africa project (GDAP) (31) (Fig. 3) will provide important resources for population and medical genetics, and also allow a clearer understanding of the evolutionary demographic history of different populations by better delineation of phase (31).

Most human assemblies have involved a haploid representation of the genome, where information from the two chromosomes is collapsed into a single sequence. Generation of haplotype representations of the genome can reduce error in the final assembly, particularly in the case of segmental duplications (16,32). While long-read technologies can generate phase information over long contiguous segments, these methods cannot resolve phase over long regions of homozygosity or assembly gaps. Assembly of haploid genomes, therefore, requires additional contextual information, which can be provided by linked-read approaches. More recently, trio based methods (where parents are sequenced using Illumina short reads, with offspring sequenced with long reads) have been used to provide this contextual information by separation of maternal and paternal haplotypes prior to assembly using a father–mother–offspring trio (33). This method has been applied to yield a highly contiguous diploid assembly of an F1 hybrid of two bovine subspecies with a quality surpassing previous cattle reference genomes (33).

Long reads have been successfully applied to organisms with smaller genomes as well as bacteria and viruses, with the advantage that for some of these the entire genome can be spanned by a single long read (34). The Tree of Life initiative, a collaboration across multiple centres is in the process of developing high resolution reference sequences for >50 vertebrate species using a combination of long read, short read and linked-read approaches. Another leading project is the large bacterial sequencing project NCTC 3000 at the Wellcome Sanger Institute, which is using PacBio sequencing to sequence complete bacterial genomes (https://www.phe-culturecollections.org.uk/collections/nctc-3000-project.aspx). These relatively small genomes (*Escherichia coli* is for example 4.6 Mbases) can often have their chromosomes and plasmids assembled into single contigs. The construction of full and accurate assemblies of these organisms allow fine-scale phylogenies of these organisms to be constructed and is also helpful in the field of epidemiology when tracing the source of an outbreak. A recent example of this was a study where SMRT sequencing was used to identify a reservoir of antibiotic resistant plasmids within hospitals (35).

In addition to DNA sequencing, ONT sequencing has been applied to sequence RNA directly rather than relying on an intermediate cDNA step, allowing direct sequencing of RNA viruses and detection of splice variants and base modifications directly from RNA molecules. An example of this is the recent direct sequencing and assembly of the influenza A virus in a native RNA form without amplification or conversion to DNA (36).

### Targeted sequencing

From a clinical point of view targeted sequencing is an area where long reads are likely to have the greatest initial impact. In the diverse, complex and clinically relevant regions such as the histocompatibility leucocyte antigen (HLA) (37), killer cell immunoglobulin-like receptor (KIR) (38) and BRCA; and in pharmacologically relevant genes such as CYP2D6 (39,40), targeted sequencing has allowed clinicians and researchers to characterize areas of the genome which were previously inaccessible using NGS methods. In addition, where diversity is high it has become possible to call and phase variation across the entire gene. This approach has since been used to retype 126 HLA reference samples across 6 loci and is now considered a gold standard for clinical sequencing for stem cell transplants (41).

Typically, when targeting such a region, a long-range PCR reaction is used to specifically amplify the genes of interest. However recently there have been studies demonstrating the use of pulldowns and CRISPR-CAS9 to capture the region of interest with little or no amplification (42). The advantage of these reduced and non-amplification based approaches is the removal of PCR error as a factor, particularly in tandem repeats and GC rich regions (42). Additionally, in the case of CRISPR methods, capture of raw genomic material allows DNA modification information to be read.

### Transcriptomics and RNA

In addition to its many uses with DNA, long-read technology also has provided many new insights into the world of transcriptomes and ncRNA by allowing for sequencing of these full length isoforms rather than relying on the assembly of sheared NGS fragments, a method prone to a high rate of false positives and ambiguities (43). Direct sequencing of isoforms can be particularly useful in complex polyploid genomes such as the coffee plant (44), where construction of a reference transcriptome is otherwise extremely challenging. In addition to its usefulness in reference transcriptomes IsoSeq has been used in functional studies to analyse the expression of various disease-linked proteins such as TP53 in leukaemia (45).

The MinION platform has recently been used to sequence cDNA; applications of this, such as single cell sequencing of immune cells illustrates the power of such methods to examine clonal heterogeneity in gene expression and isoform usage, potentially revolutionizing our understanding of the repertoire and functions of immunological cell receptors (46).

### Epigenetics

SMRT sequencing technology is able to detect base modification, as it records base kinetics of the polymerase, when DNA molecules are sequenced directly without PCR. Similarly, Nanopore technology can also detect base modifications due to variation in ionic currents. However, because amplification of DNA would erase base modifications, these methods require relatively large amounts of native, unamplified DNA as input material. Recent innovations that combine bi-sulphate conversion with SMRT sequencing have allowed direct high throughput analysis of CpG methylation without requiring large quantities of sample (47), providing an avenue for more accurate assessment of CpG islands, and allele-specific CpG methylation.

### Clinical applications

The advantages of long-read technologies in accessing complex regions of the genome, make these ideal for clinical applications in diagnosis, prognostication and personalized medicine. Early clinical applications have included sequencing of tandem repeats in fragile X syndrome, spinocerebellar ataxia, providing accurate diagnostics and potential for prognostication in clinical genetics. SMRT sequencing has also been used to resolve structural variants associated with Mendelian disease (48).

Long-read sequencing technologies are rapidly moving towards the mainstay of high resolution HLA typing for transplant registries in certain regions (37); with high resolution typing potentially having implications for better matching, and clinical outcomes of patients undergoing transplantation. This is even more important in populations which are poorly represented in current reference sequence databases, limiting disambiguation of clinical types when using standard methods for typing. The HLA diversity in Africa project, which aims to characterize high resolution HLA types across >20 ethno-linguistic in Africa has recently completed sequencing of ∼2000 individuals using long-read sequencing, identifying high levels of novelty in class I and class II HLA types (49). This panel will provide an important resource for clinical HLA typing in populations of African ancestry, as well as a platform for highly accurate imputation of HLA types in medical genetics research.

Using long-range PCR amplicons, with barcoding and long-read technology also allow better delineation of genes from pseudogenes, such as for sequencing PKD1 for diagnosing autosomal-dominant polycystic kidney disease, for which diagnostic accuracy of NGS technologies has been limited (50). SMRT sequencing has also been used to tailor treatment in patients with cancer, by identifying low frequency resistant mutations in BCR-ABL1 that affect treatment efficacy in patients with CML (51). Applications of SMRT sequencing in reproductive medicine, to identify parent of origin effects, and for pre-implantation diagnosis have been previously noted (52).

Full sequencing of several virus genomes in a single contig by long-read sequencing has provided unique avenues for identification of resistant mutations for clinical applications. Proof-of concept studies have generated protocols to examine low frequency (up to 0.25%) associated mutations for HIV and HCV resistance to drugs, through deep sequencing of full length quasispecies (53). Methylation profiles of pathogens examined using SMRT approaches have also been shown to correlate with pathogenicity, and virulence, potentially providing a new avenue for applications in infectious disease surveillance.

## The Future

Long-read technologies are improving rapidly, and may become the mainstay of sequencing; however, the broader application of long-read technologies are currently limited by a lower throughput, higher error rate and higher cost per base relative to short read sequencing. Wider use of such technologies in the clinical context may rapidly improve our understanding of cancer, pathogen evolution, drug resistance and genetic diversity in complex regions of the genome that have important implications for clinical care. Parallel development of existing technology to allow high throughput PCR-free sequencing will be important in sequencing difficult regions of the genome (54).

At present, no single long-read technology has any clear advantage from a scientific point of view, and thus it seems likely that the future of long-read sequencing is more likely to be decided on commercial terms rather than scientific. Whichever technology captures the market, it is clear that as these technologies become more affordable they will continue to shine a light into previously intractable regions of the genome with ever larger sample sizes and longer read-lengths, allowing new discovery in these evolving fields.
